# Effects of litter input on soil aggregation and aggregate carbon turnover differ among three subtropical forests in southeastern China

**DOI:** 10.3389/fpls.2025.1516775

**Published:** 2025-06-05

**Authors:** Ya-Lin Hu, Zhi-Heng Zheng, Chu-Qiao Qin, Sebastian Leuzinger

**Affiliations:** ^1^ Forest Ecology & Stable Isotope Research Center, College of JUNCAO Science and Ecology, Fujian Agriculture & Forestry University, Fuzhou, China; ^2^ University Key Laboratory of Fujian and Taiwan Characteristic Agriculture and Forestry Ecosystem Carbon Neutral, Fujian Agriculture & Forestry University, Fuzhou, China; ^3^ Forest College, Fujian Agriculture & Forestry University, Fuzhou, China; ^4^ School of Science, Auckland University of Technology, Auckland, New Zealand

**Keywords:** aboveground litter, root litter, subtropical forest, soil aggregation, litter-derived carbon, soil native carbon

## Abstract

**Background and aims:**

Litter input plays important roles in controlling soil aggregation and aggregate carbon (C) content. However, the effects of litter input on soil aggregate C turnover in different forest types remain unclear.

**Methods:**

We examined the changes of aggregate mass proportion, and the litter-derived and native C content among soil aggregates after three years of aboveground and root litter input, using ^13^C isotope tracing in a natural forest, a Chinese fir (*Cunninghamia lanceolate*) plantation, and a masson pine (*Pinus massoniana*) plantation in southeastern China.

**Results:**

Belowground root litter rather than aboveground litter input enhanced soil aggregation. Litter input increased total C content across all aggregates, and the effects were no different between aboveground litter and belowground root litter input except for the >2 mm fraction. Belowground root litter input led to less native C content across three forest types. However, belowground root litter input resulted in more formation of litter-derived C than aboveground litter input under masson pine plantations, but not for both natural forest and Chinese fire plantation, suggesting a different effect of litter input on the litter-derived C formation among forest types. In addition, forest type affected soil aggregation and aggregate C turnover, and the differences in litter quantity and litter C:N ratio can explain the changes in soil aggregation and aggregate C turnover among forest types.

**Conclusion:**

Our results imply that belowground root litter input plays a more important role in controlling soil aggregation and aggregate C turnover than aboveground litter, and the impact on newly litter-derived C formation depends on forest type.

## Introduction

Plant litter input plays an important role in controlling soil organic carbon (SOC) stocks. During litter decomposition, litter carbon (C) can return to the atmosphere as CO_2_, or enter into soils via several pathways e.g., dissolved organic carbon leaching, litter fragments and soil microbial entombing (Cotrufo et al., 2015; Liang et al., 2017). Although the factors controlling litter decomposition rates are well understood (McKinley et al., 2011), it is still unclear what proportion of plant litter C is incorporated and stabilized in soils, versus that lost to the atmosphere (Cotrufo et al., 2015; Prescott, 2010).

In forests, plant litter can mainly be divided into aboveground litter (e.g., leaves, branches, and bark) and belowground litter (e.g., root exudates and root residues) (Freschet et al., 2013; Mambelli et al., 2011). Some studies reported that aboveground leaf litter input was important in maintaining SOC in a temperate oak forest (Zhang et al., 2023) and two subtropical *Acacia crassicarpa* and *Eucalyptus urophylla* plantations (Cao et al., 2020). However, several recent studies have suggested that belowground root litter inputs resulted in greater SOC formation than aboveground litter input (Bird and Torn, 2006; Huang et al., 2021; Mambelli et al., 2011; Rasse et al., 2005; Liu et al., 2019). The pathways of aboveground and root litter C entering the soil are different (Almeida et al., 2021; Cotrufo et al., 2015; Zhang et al., 2023), because aboveground litter is generally enriched in easily degradable compounds (e.g., soluble constituents, low C:N ratio), whereas root litter has larger fractions of less degradable components (e.g., high hydrophobicity, high C:N ratio). In addition, the change of total SOC content in bulk soils is relatively slow and difficult to measure over short periods because of the simultaneous formation of newly litter-derived C and mineralization of native C (Sokol et al., 2019). The best way to monitor SOC turnover of litter-derived SOC formation and native SOC mineralization are ^13^C isotopic tracing methods, such as the ones based on the differences of δ^13^C in C3 and C4 plants (Bird and Torn, 2006; Huang et al., 2021; Hobbie and Werner, 2004; Sayer et al., 2011).

Soil organic carbon stocks and stability are not only related to chemical recalcitrance, but also controlled by physical disconnection (Schmidt et al., 2011), including spatial inaccessibility to microbes (e.g., aggregation occlusion, hydrophobic encapsulation) and ‘matrix stabilization’ via integration of soil organic and mineral components (Cotrufo et al., 2013; Liu et al., 2019). Soil aggregation is a keystone factor determining the soil’s ability to store carbon through the physical protection of organic molecules (Six et al., 2000). Conversely, soil organic matter also strongly influences soil aggregation (Six et al., 1999; Sarker et al., 2022; Tisdall and Oades, 1982), depending on soil texture, clay mineralogy, cation content, and aluminium and iron oxides (Sarker et al., 2022). Over the past several decades, positive, negative, or neutral effects on soil aggregation have been reported from experiments using a wide range of organic matter input to soils (Abiven et al., 2009; Sarker et al., 2022). The effects of organic inputs on soil aggregation depend on the quantity and quality of organic matter input (Tisdall and Oades, 1982; Abiven et al., 2009; Mizuta et al., 2015; Sarker et al., 2022). To date, the changes in soil aggregation and aggregate C in response to litter quantity and quality have been well studied in cropland ecosystems (Abiven et al., 2009; Gentile et al., 2011; Helfrich et al., 2008; Mizuta et al., 2015; Morris et al., 2019; Rillig et al., 2015; Sarker et al., 2022). However, only few studies have focused on the changes in soil aggregation and aggregate C content relative to litter input in forests (Almeida et al., 2021; Shi et al., 2023).

To understand the relative effects of aboveground and root litter input on soil aggregate C turnover among different forests, we carried out a 3-year experiment using sugarcane (C4 plant) cropland soil in three types of forest (C3 plants): natural evergreen broad-leaved forest, Chinese fir (*Cunninghamia lanceolate* (Lamb.) Hook.) plantation and masson pine (*Pinus massoniana* Lamb.) plantation in a subtropical region in southeastern China. The evergreen broad-leaved forest represents the typical local vegetation, and Chinese fir and masson pine plantations are the largest two artificial forests in subtropical region in China. We determined the mass proportion of soil aggregates, aggregate-associated organic C content, and δ^13^C, in order to infer the newly litter-derived C and native C contents. Furthermore, we examined correlations between litter quantity and quality (i.e., the C:N ratio) and litter-derived C and native C in each aggregate fraction. First, we hypothesized that the effect of root litter on soil aggregation is greater than that of aboveground litter, as root and fungal hyphae are the main binding agents that hold soil particles together (Rillig et al., 2015). Second, we hypothesized that the input of root litter induces greater litter-derived C accumulation and native C loss across soil aggregate fractions relative to aboveground litter, and soil aggregate C turnover varies between forest types due to different litter quantity and quality (Almeida et al., 2021; Zhang et al., 2023).

## Materials and methods

### Study site

Our study was conducted at the Xiqin Forest Farm of Fujian Agriculture and Forestry University (N26°34′25.43″, E118°06′44.30″), Nanping city, Fujian Province, China. The climate is subtropical monsoonal, with a mean annual temperature (MAT) of 18.2°C and mean annual precipitation (MAP) of 1860 mm. The soil is classified as red soil according to the Chinese Soil Classification System, equivalent to an Ultisol in the United States Department of Agriculture (USDA) Soil Taxonomy classification system.

### Soil transplantation experiment

The C3/C4 transplantation experiment was used to trace soil organic carbon turnover between the litter-derived C formation and soil native C retention. Before the transplantation experiment, we collected C4 soils from a cropland being used for sugarcane (C4 plant) for more than twenty years at the experimental farm of Fujian Agriculture and Forestry University, located at Fuzhou in southeastern China. The sugarcane cropland soil is a loam soil with 40.3% sand, 42.6% slit and 17.1% clay, and a pH of 5.13. Before the transplantation experiment, we randomly collected surface mineral soil from a depth of 0–20 cm, and removed all plant residues and roots, then air dried the soil and passed it through a 3 mm mesh sieve for bulk soil mixing.

We used a split-plot experimental design. First, we selected three forest types of natural evergreen broad-leaved forest (NF), Chinese fir plantations (CP), and Masson pine plantation (MP) with four replicated stands. The dominant tree species of the natural forest were *Altingia gracilipes, Aidia cochinchinensis, Cyclobalanopsis pachyloma* and *Machilus velutina*, more detailed information on stand location and litter quantity and quality are shown in **Supplementary Table S1**. One plot was set up in each stand, and conducted four litter input treatments, including aboveground litter input (AL), belowground litter input (BL), and aboveground plus belowground litter input (AL+BL), and no litter input (NL) as a control (**Supplementary Figure S1**). For the litter input treatments, four holes (6 cm diameter, 20 cm depth) were drilled using a soil auger, and the previously collected sugarcane soils (C4) were filled into the collars to replace the forest soils (C3). For NL treatment, the belowground root was prevented to grow into soils by the PVC collars, and the aboveground litter was also prevented from falling onto the soils by covering a coarse high-density nylon net (mesh size: 1 mm × 1 mm). For the AL treatment, the belowground root was prevented to grow into soils by the PVC collars, whereas the forest floor litter was placed back onto the soil surface and fresh aboveground litter was allowed to fall onto the soils. For the BL treatment, roots were allowed to grow into soils through the nylon net collars, but the fresh aboveground litter was prevented from falling onto the soils. For the AL+BL treatments, roots were allowed to grow into soils through the nylon net collars, and the forest floor litter was placed back onto the soil surface and fresh aboveground litter was allowed to fall onto the soils. In this study, the leaching of C from the tree canopy and the potential C input from belowground root exudates were not considered as a minor C input to soils. In each stand, four groups of litter input treatments were set up to collect soil samples after 6, 12, 24 and 36 months.

### Measurement of aboveground and belowground litter input

For measuring the quantity and quality of aboveground and belowground root litter input, we used three 50 cm× 50 cm litter fall traps in each stand to collect the aboveground litter falling from the tree canopy during April 2017 to April 2018. For the belowground root litter, we collected three root samples using soil augers (diameter 5 cm) in 0–20 cm profiles in each stand. The litter samples were dried at 60°C for 72 h. Then the samples were ground with a ball mill to determine the C and N content, as well as δ^13^C using an Elemental Analyzer (Vario Micro cube, Elementar, Germany) interfaced with an isotope ratio mass spectrometer (Isoprime100, Elementar, Germany).

### Fractionation of water stable aggregates, and aggregate C and δ^13^C analyses

After 6, 12, 24 and 36 months, one group of soil samples was taken back to lab for analysis. Plant residues were removed by hand, and soils were air dried. The fractionation of water stable aggregates was determined using a modified wet-sieving procedure suggested by Six et al. (1999). Briefly, 50 g of air-dried bulk soil was placed on the top of a set of nested sieves (5 mm, 2 mm, 1mm, 0.5 mm, 0.25 mm and 0.053 mm). Samples were soaked in deionized water for 15 minutes to allow slaking of unstable aggregates. Following this, the nested sieves were gently oscillated (3.5cm amplitude of 35 strokes min^-1^) within a column of water for 30 min. The smallest fraction (<0.053 mm) was recovered after evaporation of the water in containers. Soil aggregate fractions on each sieve were washed into aluminium trays, and dried at 105°C to constant weight for calculation of the mass proportion of each aggregate fraction.

Soil aggregate fractions were ground using a ball mill, and C content and isotopic abundance (δ ^13^C) were determined using an Elemental Analyzer (Vario Micro cube, Elementar, Germany) interfaced with an isotope ratio mass spectrometer (Isoprime100, Elementar, Germany). Precision of measurements was 0.1‰ for δ^13^C, and three standards of L-histidine, D-glutamic acid and glycine were used for data calibration.

### Data calculation and statistical analyses

Mass proportion of each soil aggregate was calculated as Equation (1):


(1) $$\text{MP}=\left({\text{W}}_{\text{fraction}}/{\text{W}}_{\text{total}}\right)\times 100\%$$

where MP (%) is the mass proportion of each aggregate, W_fraction_ and W_total_ are the respective masses of each fraction and the combined aggregates.

The fraction factor of litter-derived C in each aggregate was calculated according to a two-source mixing model for the difference in δ^13^C value between the litter-treated aggregate (*δ* ^13^C_soil_) and the initial sugarcane soil aggregate (δ^13^C_ini_) as Equation (2) (Balesdent and Balabane, 1996):


(2) $$\text{F}=\;\left(\delta^{13}{\text{C}}_{\text{soil}}-\delta^{13}{\text{C}}_{\text{ini}}\right)/\left(\delta^{13}{\text{C}}_{\text{lit}}-\delta^{13}{\text{C}}_{\text{ini}}\right)$$

where F is the fraction factor of litter-derived C content in soils, which ranges from 0 to 1. No litter-derived C is assumed to be contained in the soils when F is 0, and all soil C is assumed to be newly derived from litter C when F is 1. In addition, *δ* ^13^C_lit_ is the value of *δ* ^13^C in aboveground and/or belowground litter.

Litter-derived C content and soil native cropland C content in each aggregate were calculated according to the following Equation (3) and (4) (Gentile et al., 2011):


(3) $${\text{SC}}_{\text{litter}-\text{derived}}=\text{F}\times {\text{SC}}_{\text{total}}$$

(4) $${\text{SC}}_{\text{native}}=\left(1-\text{F}\right)\times {\text{SC}}_{\text{total}}$$

where SC_litter-derived_ (g kg^-1^ aggregate) is the newly transformed litter C into soil aggregate, and SC_native_ (g kg^-1^ soil) is the retained native C content. SC_total_ is the total C content in each aggregate (g kg^-1^ aggregate).

The statistical analyses were performed using the R statistical software (version 4.0.1), and the graphs were prepared using OriginPro 2021 (OriginLab Corporation, Massachusetts, USA). We tested for homogeneity of variance and the normal distribution of data. Thereafter, a repeated measures ANOVA was performed to examine the differences among litter input as a major effect, and forest types and times, and all aggregate fractions were tested separately, followed by Tukey’s HSD tests for pairwise comparisons. Pearson’s correlation analysis was used to test the relationships between litter quantity and quality, and aggregate mass proportion, total, litter-derived and native C contents. We defined *p*<0.05 as the minimal significance level.

## Results

### Aboveground and root litter quantity and quality

**Figure 1:**
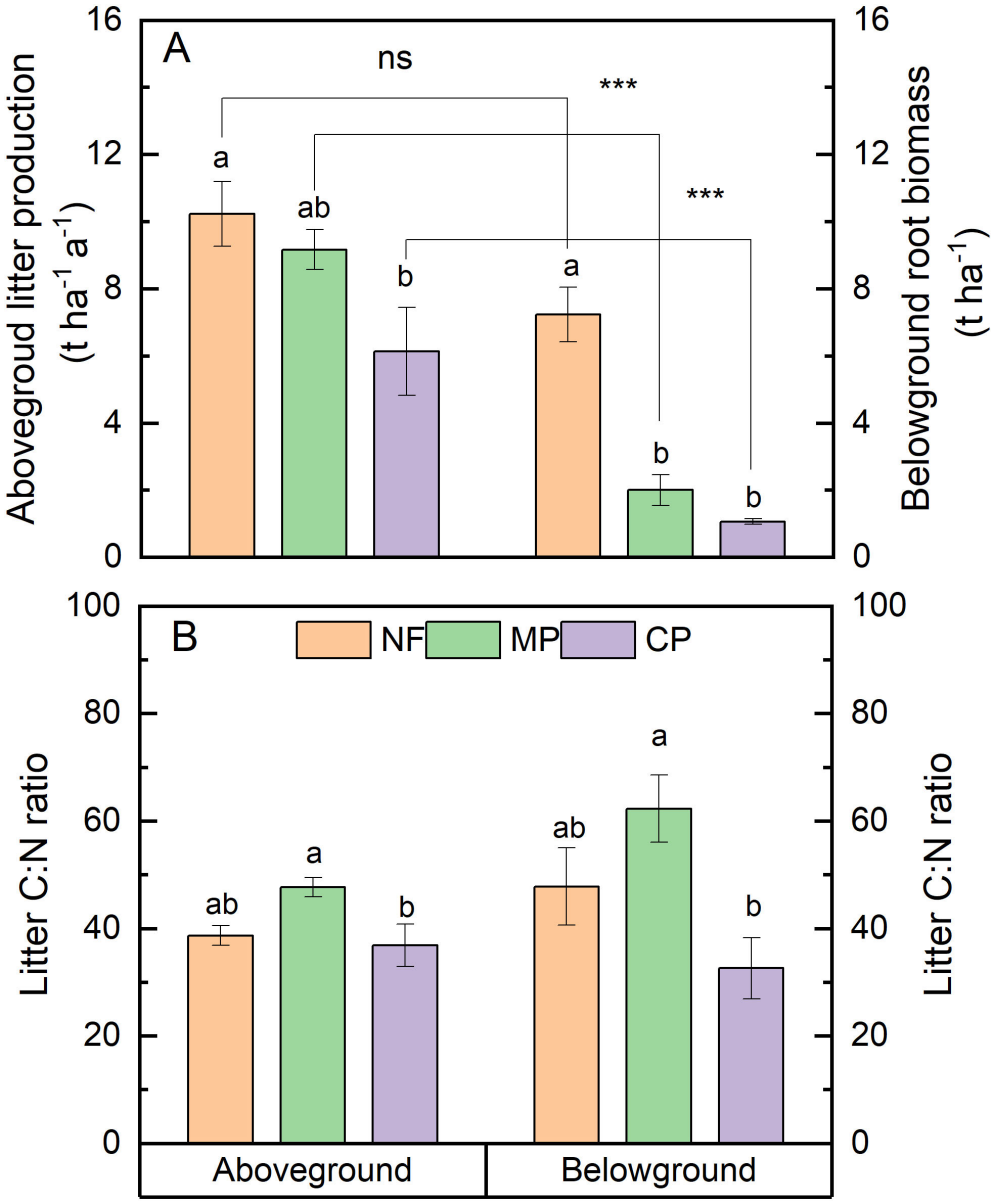
Differences of **(A)** litter quantity and **(B)** litter C:N ratio of aboveground litter and belowground root litter under three forest types. CP, Chinese fir plantation; MP, masson pine plantation; NF, natural forest. The different lowercase letters indicate significant differences among forest types at a level of p<0.05. *** indicate significant differences among between aboveground and belowground litter at a level of p<0.001. ns, no significant differences between aboveground and belowground litter.

The aboveground litter production differed from the belowground root biomass except for natural forest, and decreased in the order of NF>MP>CP (**Figure 1A**). Litter C:N ratios were not different between aboveground litter and belowground litter (**Figure 1B**). However, the masson pine stands showed higher C:N ratios of both aboveground and belowground litter as compared to Chinese fir stands.

### Soil aggregate mass proportions

**Figure 2:**
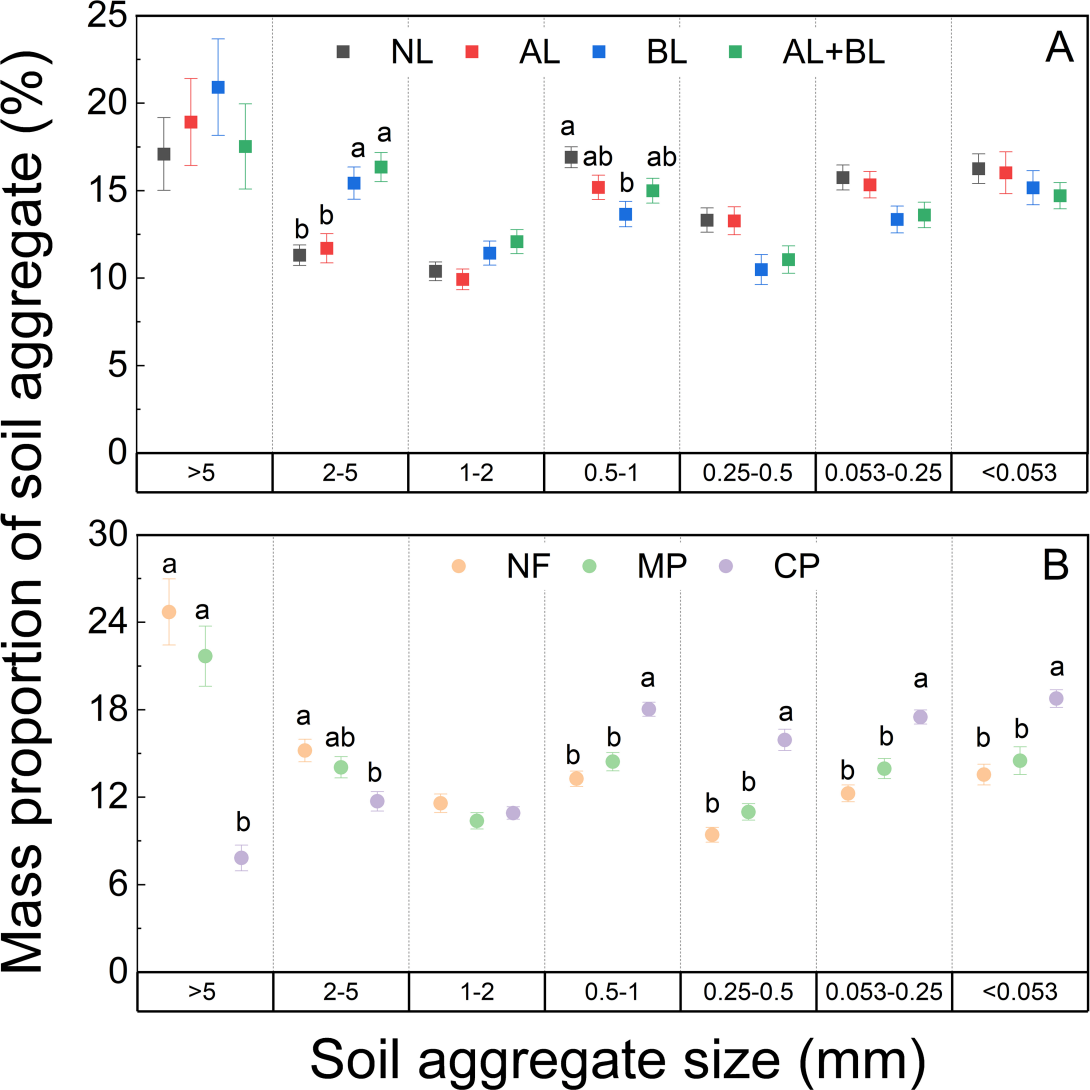
Changes of soil aggregate mass proportion induced by **(A)** litter input and **(B)** forest type. NL, no litter input; AL, aboveground litter input; BL, belowground root litter input; AL+BL, aboveground plus belowground litter input. CP, Chinese fir plantation; MP, masson pine plantation; NF, natural forest. The different lowercase letters indicate significant differences among treatments at a level of p<0.05.

The mass proportion of soil aggregates were significantly affected by litter inputs, except for the >5 mm and <0.053 mm fractions (**Supplementary Table S2**). Belowground root litter inputs enhanced soil aggregation, with the higher mass proportion of 2–5 mm fraction in both BL and AL+BL treatments (**Figure 2A**). But the mass proportions of 0.5–1 mm fraction were lower in BL than NL. Moreover, soil aggregate mass proportions significantly differed among forest types (**Supplementary Table S2**). The mass proportions of >2 mm fractions increased in the order of CP<MP<NF, while that of the <1 mm fractions were opposite (**Figure 2B**).

### Total C and δ^13^C content of the different aggregate fractions

**Figure 3:**
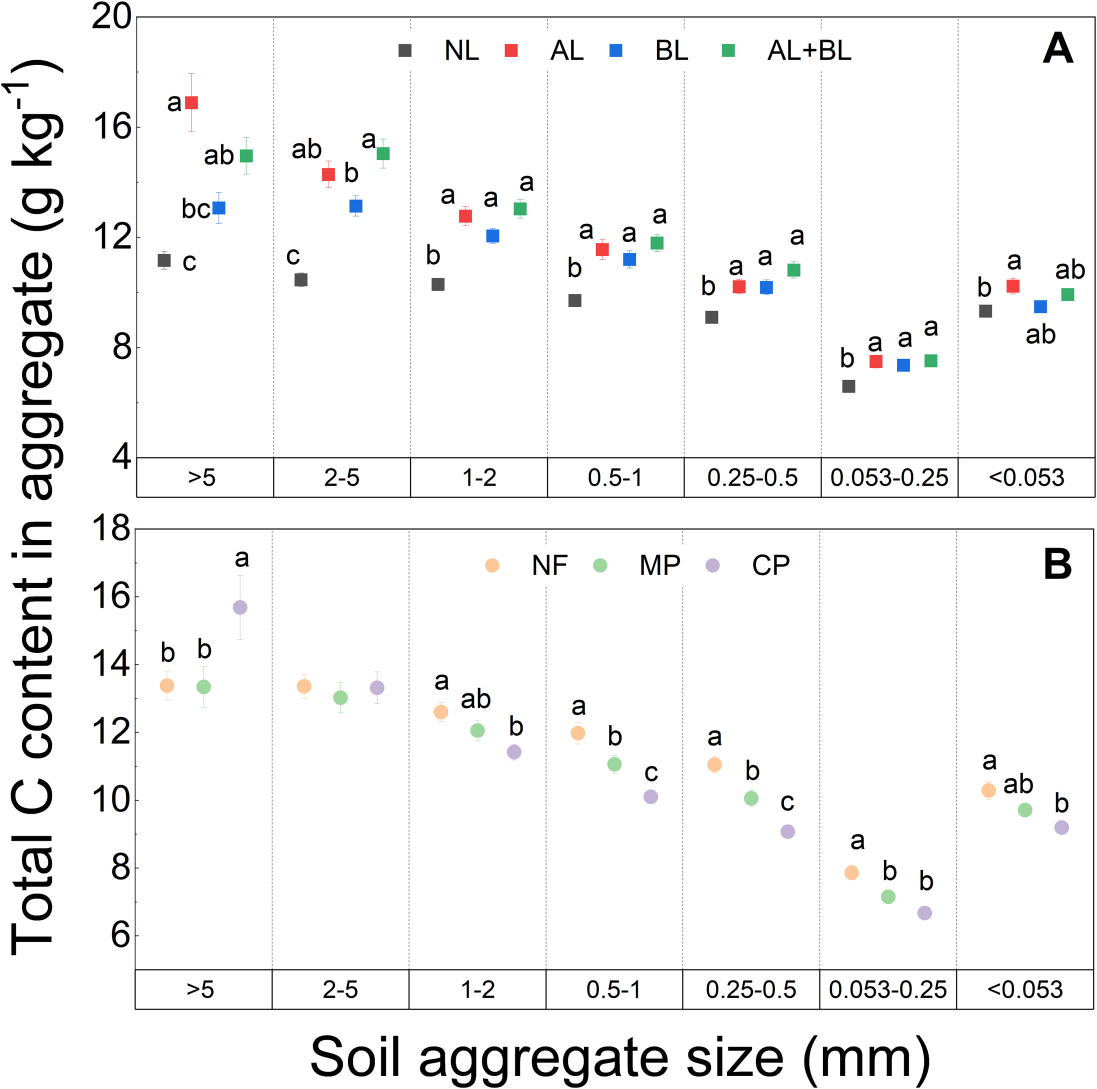
Effects of **(A)** litter input and **(B)** forest type on total C content of each aggregate fraction. NL, no litter input; AL, aboveground litter input; BL, belowground root litter input; AL+BL, aboveground plus belowground litter input. CP, Chinese fir plantation; MP, masson pine plantation; NF, natural forest. The different lowercase letters indicate significant differences among treatments at a level of p<0.05.

There were no interactive effects of litter inputs and forest types on the total C content of all aggregate fractions (**Supplementary Table S2**). Compared to the NL, the total C content of each aggregate fraction was higher with litter inputs (**Figure 3A**). Whereas there were no significant differences in total C content among litter input treatments, except for the higher total C content of the >5 mm fraction in AL than BL, and the higher total C content of the 2–5 mm fraction in AL+BL than BL. Among forest types, the total C content of the < 2 mm fraction followed the order of CP<MP<NF, while that of the >5 mm fraction in both NF and MP was lower than CP (**Figure 3B**).

**Figure 4:**
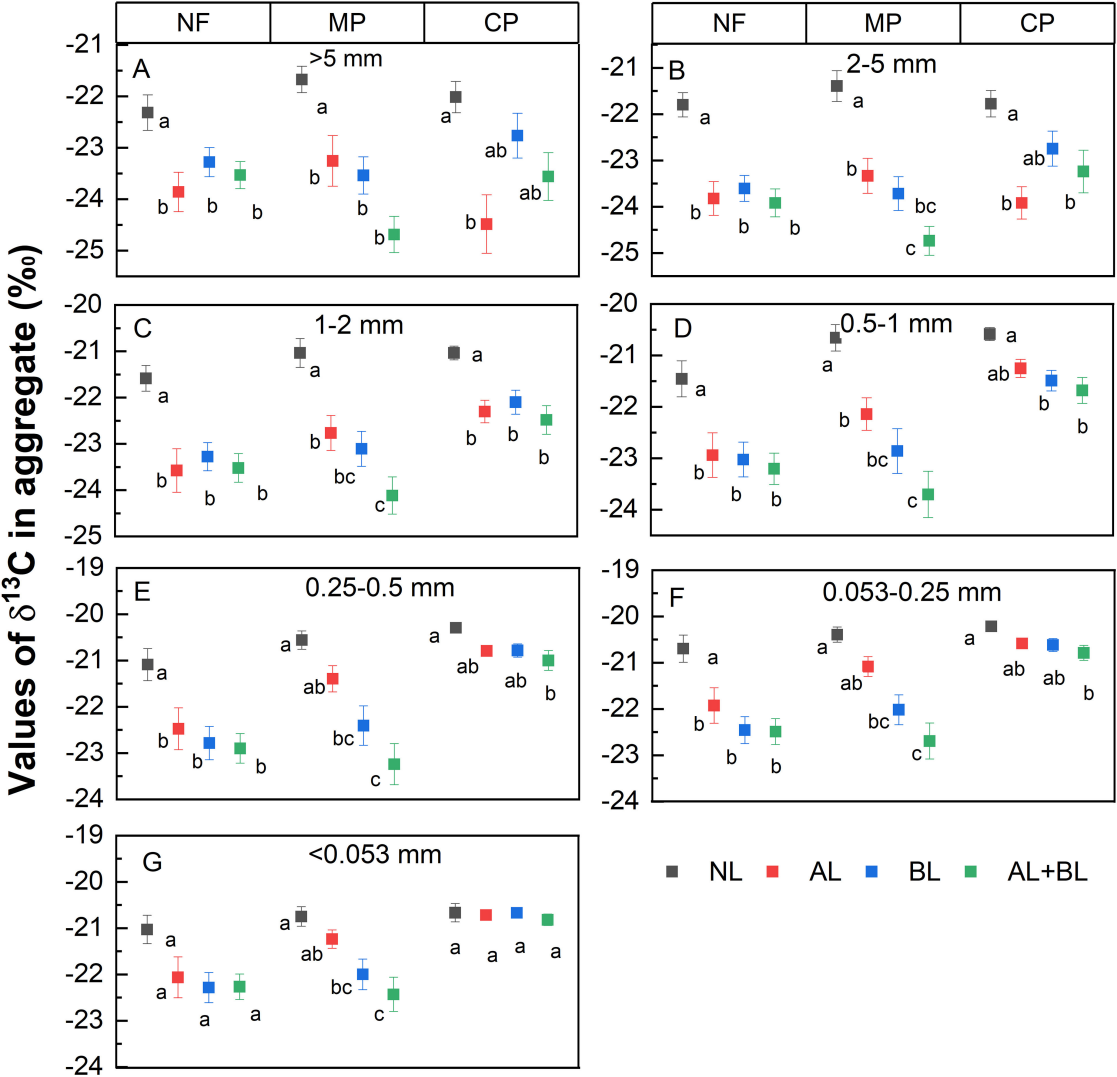
Effects of litter input on the values of δ^13^C of **(A)** >5 mm, **(B)** 2-5 mm, **(C)** 1-2 mm, **(D)** 0.5-1mm, **(E)** 0.25-0.5 mm, **(F)** 0.053-0.25 mm, **(G)** <0.053 mm aggregate fraction under three forest stands. NL, no litter input; AL, aboveground litter input; BL, belowground root litter input; AL+BL, aboveground plus belowground litter input. CP, Chinese fir plantation; MP, masson pine plantation; NF, natural forest. The different lowercase letters indicate significant differences among litter input treatments under three forest types at a level of p<0.05, respectively.

Litter input had significant influences on the *δ* ^13^C values of soil aggregates, which varied among forest types except for the >5 mm fraction (**Supplementary Table S2**). Compared to the NL treatment, litter inputs depleted the *δ* ^13^C of all aggregates (**Figure 4**). For MP, the *δ* ^13^C values of all fractions showed clear declining trends in the order of NL>AL>BL>AL+BL. However, the *δ* ^13^C values were not significantly different among AL, BL and AL+BL treatments in both NF and CP. In addition, the averaged *δ* ^13^C across litter inputs differed significantly among forest types with lower *δ* ^13^C values in both NF and MP than CP, except for > 2 mm fractions.

### Litter-derived C and native C content of the different aggregate fractions

**Figure 5:**
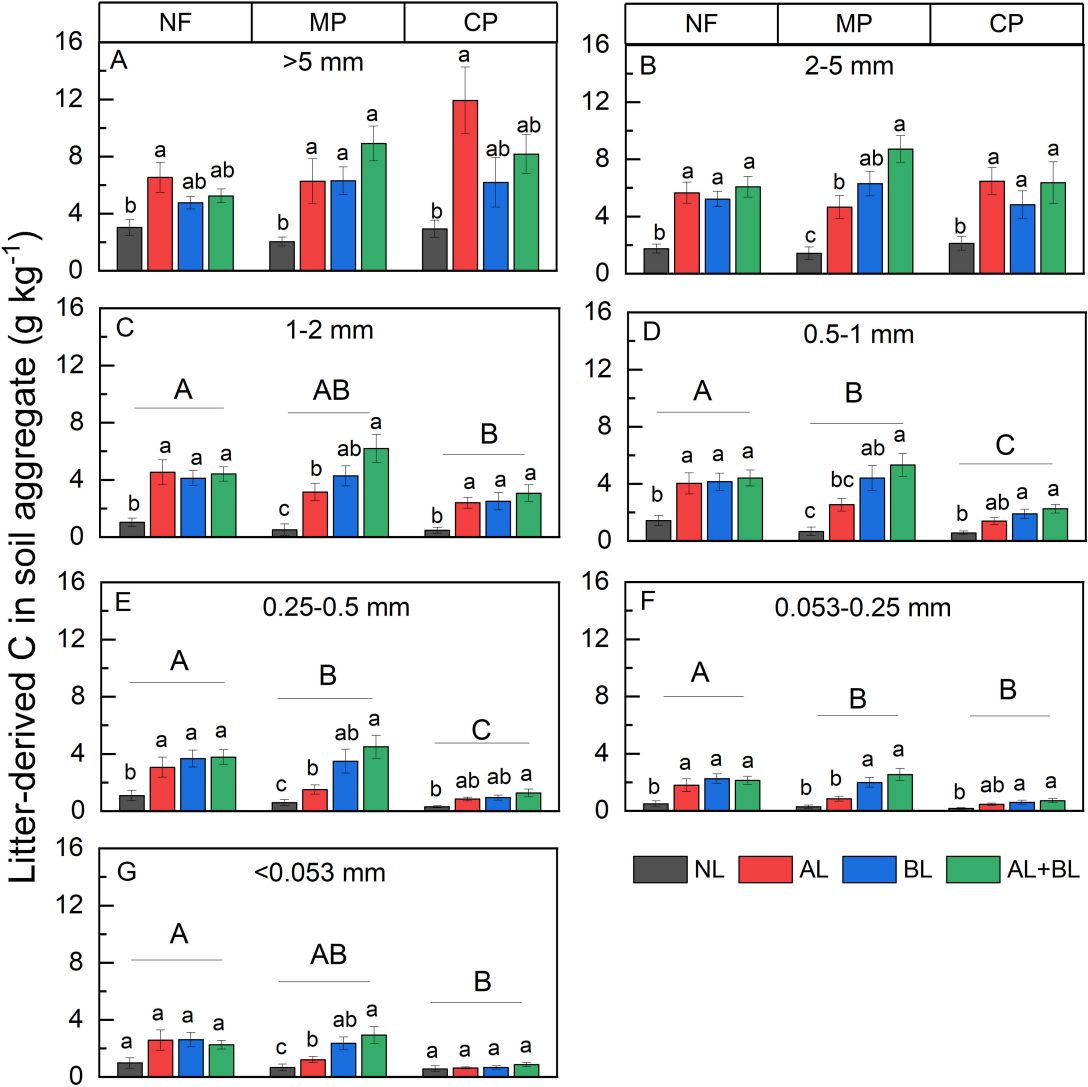
Effects of litter input on litter-derived C content of δ^13^C of **(A)** >5 mm, **(B)** 2-5 mm, **(C)** 1-2 mm, **(D)** 0.5-1mm, **(E)** 0.25-0.5 mm, **(F)** 0.053-0.25 mm, **(G)** <0.053 mm aggregate fraction under three forest stands. NL, no litter input; AL, aboveground litter input; BL, belowground root litter input; AL+BL, aboveground plus belowground litter input. CP, Chinese fir plantation; MP, masson pine plantation; NF, natural forest. The different lowercase letters on bars indicate significant differences of among litter input treatments at a level of p<0.05. The different capital letters on bars indicate significant differences of averaged litter-derived C content among forest types at a level of p<0.05, respectively.

The effect of litter input on the litter-derived C content varied with forest type (**Supplementary Table S3**; **Figure 5**). Litter input generally increased litter-derived C content as compared to the NL treatment. Moreover, the litter-derived C content in the AL treatment was generally lower than in the AL+BL treatment under MP (**Figures 5A–G**), but not for NF and CP. In addition, forest type had a significant effect on the mean of litter-derived C content of < 2 mm fractions that clearly decreased in the order of NF > MP> CP (**Figures 5C-G**).

**Figure 6:**
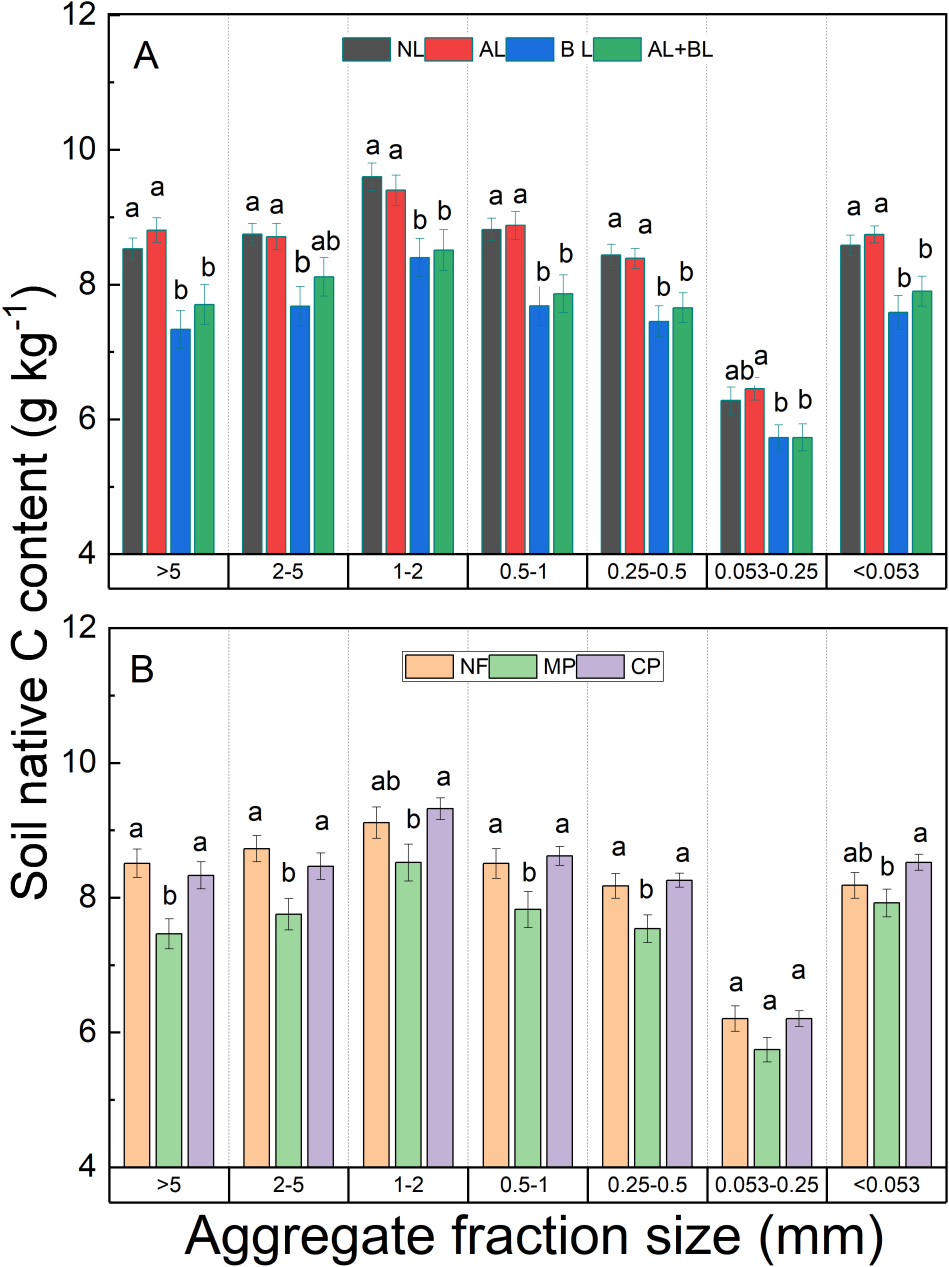
Effects of **(A)** litter input and **(B)** forest type on native C content of each aggregate fraction. NL, no litter input; AL, aboveground litter input; BL, belowground root litter input; AL+BL, aboveground plus belowground litter input. CP, Chinese fir plantation; MP, masson pine plantation; NF, natural forest. The different lowercase letters indicate significant differences among treatments at a level of p<0.05.

Native C content was significantly influenced by litter input and forest type, but there was no interactive effect between litter input and forest type except for the 0.25-0.5 mm fraction (**Supplementary Table S3**). Across all fractions, the belowground root litter input (i.e., BL and AL+BL) had lower native C content compared to NL and AL (**Figure 6A**). Among forest types, the native C content under MP was lower than both CP and NF (**Figure 6B**).

## Discussion

### Aboveground and belowground litter differently affect soil aggregation

We observed that belowground root litter input enhanced soil aggregation, but not for aboveground litter input. Our results indicated that belowground root litter input has a greater effect on soil aggregation than aboveground litter input, supporting our first hypothesis. The enhanced soil aggregation induced by belowground root litter input might be related to several mechanisms. Firstly, plant roots and their mycorrhizal symbionts can directly increase organic binding agents such as root, hyphae, and polysaccharides that drive individual mineral particles to be held together to form macroaggregates (Tisdall and Oades, 1982; Siddiky et al., 2012; Morris et al., 2019; Rillig et al., 2015). Secondly, the belowground root residues and exudates can alter soil microbial communities (Jing et al., 2021), which possibly are involved in soil aggregation processes (Lehmann et al., 2017; Laub et al., 2022; Ortiz et al., 2022). Furthermore, some previous studies suggested that arbuscular mycorrhizal fungi play an important role in the fungal energy channels of the soil food web, and alter the feces of soil fauna that could contribute to soil aggregation by serving as starting nuclei for soil aggregates (Siddiky et al., 2012). In addition, root physical penetration altered the soil structure, such as pore-clogging, compression, macro-aggregate cracking, root shrinkage-induced preferential channels, and micro-aggregate amalgamation (Xiao et al., 2024).

### Turnover of litter-derived and native C induced by litter inputs

In our study we found that the inputs of aboveground and/or belowground root litter lead to increasing total C content of each aggregate fraction compared to the control, and which did not vary among forest types. Consistently, many previous studies observed soil aggregate C content increased by organic matter inputs (Almeida et al., 2021; Sarker et al., 2022; Zhang et al., 2023). A meta-analysis of global litter-manipulation experiments reported that litter addition increased total carbon in the mineral soil by 10%, despite higher rates of carbon release (Xu et al., 2013). However, there were no differences in total C contents between aboveground and belowground litter, and which was not consistent among previous studies showing the greater effect of root litter on soil C (Bird and Torn, 2006; Huang et al., 2021; Mambelli et al., 2011; Rasse et al., 2005; Liu et al., 2019). Moreover, several previous studies suggested a more important role in the formation of SOC induced by aboveground litter input (Cao et al., 2020; Xu et al., 2021). In this present study we observed higher total C content of the > 5 mm fraction caused by aboveground litter input rather than root litter (**Figure 3a**), but not for the other aggregate fractions. It has been suggested that aboveground litter promotes net C gains in both particulate organic carbon (POC) and mineral-associated organic carbon (MAOC), whereas root litter only led to net C gains in POC but not the total SOC in bulk soils (Almeida et al., 2021; Zhang et al., 2023). Our results implied that the SOC formation of the large size fraction was more likely to be different between aboveground and belowground litter input.

Soil carbon pool sizes are mainly determined by the balance of newly litter-derived C formation and native C mineralization that is related to litter input (Almeida et al., 2021; Cotrufo et al., 2015; Zhang et al., 2023). Many previous studies reported the different litter-derived C formation between aboveground litter and root litter (Almeida et al., 2021; Bird and Torn, 2006; Gale et al., 2000; Shi et al., 2023; Zhang et al., 2023). Similarly, we observed that the input of belowground litter induced more litter-derived C formation than the aboveground litter under the masson pine plantation, implying that root litter had the greater contribution of the newly accumulated C within aggregates than aboveground litter (Shi et al., 2023). However, the greater litter-derived C contents induced by belowground litter input were mainly observed in the <5 mm fractions, which might be related to the more important contribution of root-derived C in stable small aggregates than surface residue-derived C (Gale et al., 2000). It has been suggested that root litter leads to greater C formation in particulate organic matter due to selective preservation of root recalcitrant components, and rhizodeposition input had greater efficiency of MAOC formation (Almeida et al., 2021; Rasse et al., 2005; Sokol et al., 2019; Villarino et al., 2021). However, some studies suggested that the formation of POC and MAOC via microbial incorporation of aboveground litter was more efficient than via belowground roots (Almeida et al., 2021; Zhang et al., 2023). In contrast to the masson pine plantation, the litter-derived C content of all aggregates did not differ between aboveground and belowground litter treatments under both natural forest and Chinese fir plantations. This confirms our second hypothesis that the relative effect of aboveground and belowground root litter input on the litter-derived C formation varies among forest types.

Litter input not only contributes to the buildup of soil C pools, but also affect soil microbial community composition and activity that control soil native C mineralization. In this study, the belowground root litter input (i.e., BL and AL+BL) decreased native C content of all aggregates under three forest types, compared to NL and AL treatments. Our results are supported by Almeida et al. (2021) who also observed lower native C content in the presence of roots as compared to leaf, twig and bark litter that provide more easily metabolized nutrients and substrates, resulting in less degradation of native C due to priming (Kuzyakov et al., 2000; Cheng et al., 2014). On other hand, the simple C substrates (e.g., root exudates) can lead to a greater mineralization of native SOC than the addition of complex C substrates (e.g., plant residues) due to the mechanism of ‘stoichiometric decomposition’ (Blagodatskaya and Kuzyakov, 2008).

### Forest type affects soil aggregation and aggregate C balance

Our results showed that soil aggregation differed between forest types. This result is consistent with Wang et al. (2022), who found that the proportion of macroaggregates in broad-leaved and bamboo forests were higher than that in Chinese fir forests. Shi et al. (2023) observed that soil aggregation was improved along a secondary successional chronosequence from pioneer forests to climax forests. Soil aggregate formation and stability depend on several biotic and abiotic factors, including organic matter quantity and initial quality (Abiven et al., 2009; Mizuta et al., 2015; Sarker et al., 2022), the root morphological characteristics (Siddiky et al., 2012; Rillig et al., 2015), as well as soil mineralogy and microclimate (Laganière et al., 2011; Toriyama et al., 2015; Shi et al., 2023). In this present study, soil mineralogy should not account for the changes in soil aggregation, considering forest soils were replaced with the same C4 soils. The changes of aggregate mass proportion might be related to the different quantities of aboveground and belowground litter, as we observed declining litter quantity from natural forest to Chinese fir plantation (**Figure 1A**). It has been suggested that a higher rate and frequency of organic litter applications can improve soil aggregation (Sarker et al., 2022). Although the litter C:N ratio of Chinese fir stands was the lowest, the mass proportions of > 2 mm fractions under Chinese fir plantation were lower than under natural forest and masson pine. Similarly, Dai et al. (2019) also observed that the mean weight diameter of aggregate was lower when added biogas residue, manure, and biochar with low C:N ratios (9.1, 9.3 and 28.9) compared to straw with a high C/N ratio (64.4). High-quality litter is highly decomposable and beneficial for the synthesis of microbial by-products, which usually result in a rapid but transient increase of soil aggregation (Abiven et al., 2009; Mizuta et al., 2015). In contrast, low-quality litter can result in a moderate long-term stimulation of microbial by-products and initiate long-term aggregation (Halder et al., 2021; Sarker et al., 2022). It has been suggested that organic C compositions among different litter types can explain the varied effects on soil aggregation better than the C/N ratio, and the organic matter rich in carbohydrate C fractions tend to induce rapid but short-term effects on soil aggregation (Sarker et al., 2022).

We found that forest type differed the total, litter-derived and native C contents, and these effects varied between different aggregate fractions. Similarly, Wang et al. (2022) also reported that the aggregate C content of the <2 mm fraction under broad-leaved forest was significantly higher than under a Chinese fir plantation in China. Lyu et al. (2017) found that the C content in the microaggregates of the two coniferous plantation forests (*C. lanceolata* and *P. massoniana*) was lower than that in a secondary forest. The differences in SOC content among forest types are related to several factors, e.g. litter production and quality, soil mineral properties, stand microclimate and soil microbial composition and activity (Laganière et al., 2011; Lyu et al., 2017; Toriyama et al., 2015; Su et al., 2021). In our study, the differences in aboveground and belowground litter quantity could explain the increasing total and litter-derived C content of < 2 mm fractions in the order of CP<MP<NF. In addition, we found that the native C content was the lowest under masson pine plantation. This might be related to the lower quality (i.e., high litter C:N ratio) of aboveground and belowground litter of masson pine, supporting a ‘microbial nitrogen mining’ of native SOC induced by lower quality of exogenous organic substrates (Chen et al., 2014; Qiu et al., 2023) and root litter (Almeida et al., 2021; Cheng et al., 2014). Considering the same sugarcane cropland soils replaced under three forest stands, our results indicate that litter quantity and quality played an important role in controlling soil aggregate C turnover in all forest types.

## Conclusion

Our study clearly showed that the input of belowground root litter input rather than aboveground litter enhanced soil aggregation, and lead to decline of native C in each aggregate fraction, implying that belowground root litter plays a more important role in soil aggregation and aggregate C turnover than aboveground litter input. In addition, our results showed that forest type played an important role in soil aggregation and aggregate C turnover, with a greater potential of C sequestration in natural forest than in the two plantation forests in this subtropical region. Higher litter input promoted soil aggregation and newly accumulated C, but lower litter quality impacted negatively on soil aggregation and aggregate C turnover among forest types. Furthermore, there was a pronounced interactive effect of litter input and forest type on the litter-derived C content, suggesting the effect of litter input on litter-derived C formation depends on forest types. In the future, the connection between litter quality and forest type should be included in a framework that considers the changes of aggregate C turnover and stability following litter input among different forest types.

## Data Availability

The raw data supporting the conclusions of this article will be made available by the authors, without undue reservation.
