# Supplementary material for: Effects of litter input on soil aggregation and aggregate carbon turnover differ among three subtropical forests in southeastern China
Source: Front Plant Sci. 2025 Jun 5;16:1516775. doi: 10.3389/fpls.2025.1516775 (PMC12178240; doi:10.3389/fpls.2025.1516775)

Table S1 Stands Characteristics and the quantity and quality of aboveground litter and belowground root litter

| Plot | Latitude /  longitude | Age  (a) | DBH  (cm) | Tree height  (m) |  | Altitude  (m) | Slope  (°) | Slope aspect |  | Aboveground litter | | | | |  | Belowground litter | | | | |
| --- | --- | --- | --- | --- | --- | --- | --- | --- | --- | --- | --- | --- | --- | --- | --- | --- | --- | --- | --- | --- |
|  |  |  |  |  |  |  |  |  |  | Litter production  (t ha^-1^a^-1^) | C  (g kg^-1^) | N  (g kg^-1^) | C:N | δ^13^C  (‰) |  | Root biomass（t ha^-1^） | C  (g kg^-1^) | N  (g kg^-1^) | C:N | δ^13^C  (‰) |
| NF1 | 26°33′27.7″N  118°06′39″E | / | 6.04 | 6.03 |  | 127.7 | 30 | Southeast |  | 10.45 | 488 | 12.88 | 37.88 | -30.17 |  | 7.23 | 401 | 13.08 | 30.69 | -28.30 |
| NF2 | 26°33′27.1″N  118°06′41″E | / | 6.44 | 6.47 |  | 160.6 | 30 | Southeast |  | 12.72 | 486 | 14.27 | 34.03 | -30.90 |  | 6.85 | 458 | 9.34 | 49.06 | -28.46 |
| NF3 | 26°33′26.8″N  118°06′41″E | / | 4.59 | 6.51 |  | 165.7 | 29 | Southeast |  | 8.14 | 488 | 11.43 | 42.72 | -30.46 |  | 5.45 | 480 | 10.53 | 45.56 | -29.36 |
| NF 4 | 26°33′25.3″N  118°06′44″E | / | 5.27 | 6.27 |  | 160.7 | 30 | Southeast |  | 9.58 | 500 | 12.51 | 40.00 | -30.90 |  | 9.38 | 468 | 7.11 | 65.80 | -29.38 |
| CP1 | 26°34′52.6″N  118°04′56″E | 22 | 22.84 | 18.24 |  | 130.6 | 28 | South |  | 4.88 | 496 | 12.29 | 40.34 | -29.13 |  | 1.20 | 444 | 11.76 | 37.76 | -26.79 |
| CP2 | 26°34′51.7″N  118°04′55″E | 22 | 23.77 | 20.52 |  | 135.4 | 29 | South |  | 5.87 | 497 | 12.44 | 39.91 | -29.19 |  | 0.92 | 450 | 9.78 | 46.02 | -26.53 |
| CP3 | 26°34′58.8″N  118°04′35″E | 16 | 16.64 | 14.35 |  | 135.4 | 29 | East |  | 9.88 | 484 | 11.53 | 41.98 | -29.45 |  | 1.22 | 455 | 17.74 | 25.64 | -28.25 |
| CP4 | 26°34′58.8″N  118°04′33″E | 15 | 20.63 | 16.45 |  | 183.9 | 30 | Northwest |  | 3.90 | 494 | 19.68 | 25.08 | -29.50 |  | 0.91 | 417 | 19.97 | 20.89 | -30.11 |
| MP1 | 26°34′47.1″N  118°02′32″E | 18 | 20.66 | 16.41 |  | 130.6 | 28 | South |  | 10.72 | 504 | 11.71 | 43.07 | -29.55 |  | 2.25 | 477 | 8.01 | 59.56 | -28.88 |
| MP2 | 26°34′47.0″N  118°02′38″E | 19 | 18.14 | 15.55 |  | 135.4 | 29 | South |  | 8.82 | 504 | 10.17 | 49.50 | -30.02 |  | 3.17 | 483 | 7.65 | 63.11 | -28.85 |
| MP3 | 26°35′24.4″N  118°00′43″E | 15 | 18.75 | 15.35 |  | 132.5 | 28 | Southwest |  | 7.86 | 505 | 9.84 | 51.31 | -29.27 |  | 1.50 | 477 | 6.09 | 78.36 | -26.33 |
| MP4 | 26°35′36.3″N  118°00′32″E | 15 | 19.89 | 15.01 |  | 183.9 | 30 | Southwest |  | 9.25 | 490 | 10.44 | 46.90 | -29.71 |  | 1.09 | 413 | 8.57 | 48.17 | -27.99 |

NF, natural forest; CP, Chinese fir plantation; MP, masson pine plantation; DBH, diameter breast height

Table S2 Repeated measures ANOVA of litter input, forest type and time on the mass proportion, total C content and δ^13^C values of each aggregate fraction.

|  | Mass proportion (%) | | |  | Total C content (g kg^-1^) | | |  | δ^13^C values | | |
| --- | --- | --- | --- | --- | --- | --- | --- | --- | --- | --- | --- |
|  | Litter input  (L) | Forest type  (F) | L ×F |  | Litter input  (L) | Forest type  (F) | L ×F |  | Litter input  (L) | Forest type  (F) | L ×F |
| >5mm | ns | *** | ns |  | *** | * | ns |  | *** | ns | ns |
| 2-5mm | *** | * | ns |  | *** | ns | ns |  | *** | ns | * |
| 1-2mm | ** | ns | ns |  | *** | ** | ns |  | *** | *** | * |
| 0.5-1mm | ** | *** | ns |  | *** | *** | ns |  | *** | *** | * |
| 0.25-0.5mm | * | *** | ns |  | *** | *** | ns |  | *** | *** | * |
| 0.053-0.25mm | * | *** | ns |  | * | *** | ns |  | *** | *** | ** |
| <0.053mm | ns | *** | ns |  | * | *** | ns |  | *** | *** | * |

*, ** and *** indicated the significant difference among treatments at the level of p<0.05, p<0.01 and p<0.001, respectively. ns, no significant difference.

Table S3 Repeated measures ANOVA of litter input, forest type and time on the litter-derived C and native C content of each aggregate fraction.

| Soil aggregate size | Litter-derived C content (g kg^-1^) | | |  | Native C content (g kg^-1^) | | |
| --- | --- | --- | --- | --- | --- | --- | --- |
|  | Litter input (L) | Forest type  (F) | L ×F |  | Litter input (L) | Forest type  (F) | L ×F |
| >5mm | *** | ns | ns |  | *** | *** | ns |
| 2-5mm | *** | ns | ns |  | *** | *** | ns |
| 1-2mm | *** | *** | * |  | *** | ** | ns |
| 0.5-1mm | *** | *** | ** |  | *** | ** | ns |
| 0.25-0.5mm | *** | *** | ** |  | *** | *** | * |
| 0.053-0.25mm | *** | *** | ** |  | *** | * | ns |
| <0.053mm | *** | *** | * |  | *** | * | ns |

**Fig. S1** Location of (a) study site and (b) forest stands, and (c) method of litter input. NL, without aboveground and belowground litter input; AL, aboveground litter input; BL, belowground root litter; AL+BL, aboveground plus belowground litter input


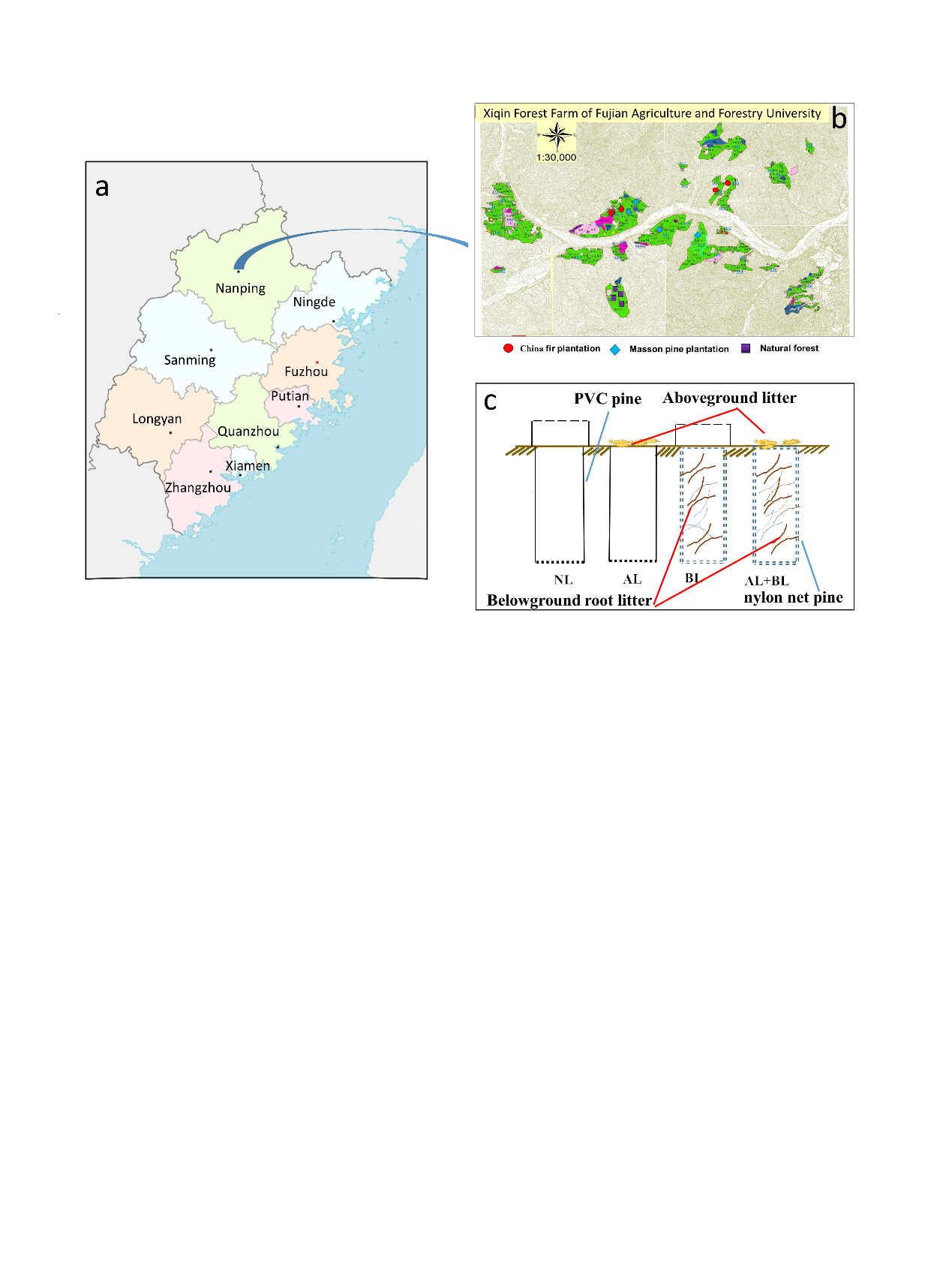

Supplement: Supplementary file 1 [file Table1.docx]
